# Finding meaning in chronic illness and its relationship to psychological well-being: A mixed-methods study

**DOI:** 10.1371/journal.pmen.0000121

**Published:** 2024-09-05

**Authors:** Rebecca J. Purc-Stephenson, Rachel Edwards

**Affiliations:** 1 Department of Social Science, Augustana Faculty, University of Alberta, Camrose, Alberta, Canada; 2 Department of Psychology, Western University, London, Ontario, Canada; Ss Cyril and Methodius University in Skopje: Saints Cyril and Methodius University in Skopje, NORTH MACEDONIA

## Abstract

Several theories propose that health-related adversity threatens an individual’s worldviews, goals, and sense of purpose, which motivates them to search for meaning. This process is believed to lead to positive adjustment and personal growth. The purpose of our study was to (a) examine whether a health-related adversity motivated a search for meaning, (b) explore the ways individuals made sense of their health-related adversity, and (c) examine whether meaning making was related to acceptance, subjective well-being (positive affect, negative affect, satisfaction with life), and posttraumatic growth (PTG). Using a mixed-methods design, we collected data from 317 adults diagnosed with inflammatory bowel disease (IBD). Participants completed a survey online. The survey included a set of validated measures that assessed acceptance, psychological adjustment, and PTG, as well as several open-ended questions. Approximately 85% of participants reported asking *why me*? Of those who reported that they found meaning, our thematic analysis revealed five meaning-making themes: catalyst for personal growth, self-blame, divine intervention, genetics, and chance. Our profile analysis compared three meaning-making groups (i.e., found meaning, still searching, found no meaning/not searching) and revealed that individuals who found meaning reported more positive affect and perceived more PTG than individuals still searching or who had not found meaning. Our data support theories of growth that suggest meaning making plays an important role in adjusting to adversity. We discuss cognitive and narrative-type therapeutic approaches that rehabilitation counselors could apply when supporting individuals coping with chronic illnesses.

## Introduction

I used to ask ‘why’ a lot. My father has Crohn’s disease but I didn’t understand why I had to have it too. I prayed and questioned God and finally decided that ‘things happen for a reason’ and I stick by that. Today I am a nurse, which I’m not sure would have happened without my illness. I love what I do, and to top it all off, I work on a general surgery floor where about 50% of my patients suffer from something in the digestive tract. Often my own personal experience seems to help people work through theirs.

(Participant 313)

Living with a chronic illness can be extremely distressing as it disrupts life goals, confronts an individual with their own mortality, and involves physical and psychological limitations [1]. Understanding why the adversity happened and the significance it has had on one’s life is believed to be critical to adjustment [2]. One cognitive process linked to adjustment is *meaning making*, which refers to searching and making sense of adversity within existing worldviews [3, 4]. The quote above is from a woman diagnosed with Crohn’s disease from our study who described her search for meaning in her illness and the significant impact she believed the disease has had on her life. Understanding the processes that help individuals adapt to chronic illnesses is essential for developing therapeutic interventions that promote their well-being and personal growth.

### Understanding IBD

Inflammatory bowel disease (IBD) is a chronic, relapse-remittent disease that affects the gastrointestinal tract, and includes Crohn’s disease and ulcerative colitis. IBD is characterized by unpredictable flare-ups, and common symptoms include diarrhea, fatigue, abdominal pain, weight loss, weight gain, and rectal bleeding [5]. Canada has one of the highest rates of IBD in the world, affecting approximately one in every 350 persons [6]. Diagnosis can happen at any age, but most often occurs between the ages of 15 and 30. While the cause of IBD is unclear, research suggests it involves a combination of genetic and environmental factors [7].

A recent meta-analysis reported that having IBD was associated with an increased risk of anxiety and depression, with prevalence rates as high as 36% for anxiety and 28% for depression [8]. Having IBD means coping with stigmatizing and embarrassing symptoms that can negatively impact an individual’s ability to work, engage in social activities, and maintain relationships [9, 10]. Also, individuals must cope with the adverse side effects (e.g., weight gain) of medications [11], the possibility of needing surgery, an ostomy bag, and an increased risk of colon cancer [12]. As the cause of IBD is unknown, there is no cure or singular effective treatment, and the episodic symptoms can cause significant physical and psychological distress, IBD represents a suitable illness context to study meaning making and how it relates to psychological adjustment.

### Making sense of chronic illness

Understanding how individuals make sense of their illness has re-emerged in various disciplines, such as public health, medical sociology, nursing, and rehabilitation psychology. This research gives a voice to patient-reported outcomes and their lived experiences [13] and plays an important role in coping with chronic illness [4]. A challenge for researchers studying this topic is that different terms are used to describe meaning making, including sense making [14], causal attributions [15], and illness perceptions [16]. For this study, we used the term *meaning making* as it moves beyond identifying a cause for the illness to understanding the significance of the illness in one’s life [2].

Meaning making has been studied in cancer research [17, 18], and has become increasingly common in research on chronically ill populations [19] including multiple sclerosis [20, 21], type 2 diabetes [22], and mixed chronic illness samples [23, 24]. A review of 27 studies reported that the most common meaning-making themes were biological factors, character or behavioral self-blame, and chance [25]. Recent research expands upon these themes by reporting that individuals use spirituality and religion to make sense of their illness [14, 20]. For example, many individuals with multiple sclerosis believed a higher power gave them their illness to teach them a lesson or to give them a new purpose [14].

While no study has focused on meaning making among individuals with IBD, several quantitative studies examined causal attributions among IBD patients [26, 27]. According to these studies, common attributions among individuals were believing IBD was caused by stress, working too hard, or having an unhealthy lifestyle. Two qualitative studies examined issues related to meaning making [28, 29]. For example, Matini and Ogden [29] interviewed 38 individuals about their adjustment to living with IBD. The results revealed that participants tried to develop a “new normal”, whereby making sense of the illness was a dominant theme. Some believed their illness was their fault whereas others believed it was from stress. Collectively, this research shows that individuals with IBD think about the causes of their illness, but we have limited understanding of how meaning making related to their adjustment.

### Theoretical framework

According to the meaning-making model [4], individuals are influenced by *global meaning*, which are beliefs about the world, personal goals, and a subjective belief of meaning and purpose in life. In the event of a potentially stressful situation, an individual engages in a process of *situational meaning* that begins by appraising how threatening the situation is. Situations appraised as threatening create a discrepancy with one’s global meaning, which in turn produces distress and activates a search for meaning to restore a belief that the world makes sense and that they have purpose and worth. This process is considered successful when it leads to psychological adjustment, such as believing that the situation “makes sense”, acceptance, or perceived personal growth.

The meaning-making model is similar to other growth theories [30–32] in that it assumes the search for meaning is triggered by a “seismic” shattering of assumptions about the world as well as a gradual breakdown of one’s assumptive world. In the context of IBD, both processes are likely to occur. For example, a seismic shattering of the assumptive world might occur when an individual is diagnosed, whereas a gradual breakdown might occur as the individual copes with the episodic nature of the disease until a new worldview is created that incorporates the ongoing challenges associated with IBD. In either situation, meaning making is expected to facilitate rebuilding global meaning, which can in turn foster adjustment.

### Meaning making and psychological adjustment

Previous research supports the assumption that individuals who make sense of their illness report positive adjustment [4]. For example, Voth and Sirois [33] examined self-blame attributions (e.g., *If I get sick I am to blame*) and responsibility attributions (e.g., *It’s up to me to avoid unhealthy behaviors*), coping, and psychological adjustment (i.e., acceptance, helplessness, and coping efficacy) among individuals with IBD. They found that self-blame attributions were directly related to increased avoidant coping, which were related to poor adjustment. In contrast, responsibility attributions were related with less avoidant coping strategies and better adjustment. Others have found that those who believed their illness was due to stress and stress behavior (i.e., working too hard, alcohol use) reported low levels of mental and physical health [27].

The evidence that meaning making may be related to improved psychological adjustment for individuals with IBD necessitates the need to better understand this phenomenon by examining a more comprehensive set of adjustment outcomes. Research examining meaning making in other chronic illnesses commonly assessed acceptance [34], satisfaction with life [14, 21, 35], positive and negative affect [14, 36], and posttraumatic growth (PTG) [4, 19]. While these five variables reflect some of the purported outcomes in the meaning-making model, there is limited understanding how these variables relate to each other in the context of meaning making, especially for individuals with IBD.

#### Meaning making, acceptance and subjective well-being

Acceptance is an assumed outcome of the meaning-making process, yet research on meaning making and acceptance is mixed. Davis and Morgan [34] reported that participants with tinnitus who never searched for meaning were least likely to report positive changes, but they did report the greatest well-being and acceptance of tinnitus. It is possible that a search for meaning fosters personal growth but not adjustment. In terms of psychological adjustment, we used Diener’s [37] tripartite formulation of subjective well-being (SWB) that consists of life satisfaction, positive affect, and negative affect. Previous research has found that meaning making can enhance life satisfaction and decrease depressive symptoms and negative affect [14, 35, 36]. For example, Dezutter et al. [35] surveyed pain patients and found that those who had found meaning and were no longer searching for meaning represented the most optimal profile with less depressive symptoms and greater life satisfaction. Conversely, those who had not found meaning and were still searching reported more depressive symptoms and less life satisfaction.

#### Meaning making and posttraumatic growth

PTG refers to positive life changes following a significant life crisis or trauma [32]. PTG is the product of a cognitive process involving a schema change due to disrupted assumptive beliefs rather than the characteristics of the event. A meta-analysis of 103 studies on PTG following traumatic events, such as having cancer or multiple sclerosis, found that positive reappraisal coping (i.e., finding personally relevant positive meaning from a negative experience) related to PTG [38]. More recently, Zeligman et al. [24] surveyed adults with a chronic illness and found that the presence of meaning was a stronger predictor of PTG compared to social support. While research has demonstrated that individuals with IBD experience PTG [10], no research has examined meaning making in relation to PTG for this patient population.

### Purpose of the study

Research on how individuals with IBD adapt to their illness is limited, and the influence of meaning making among this patient population has not been explored despite calls for researchers to investigate meaning making for individuals with chronic illnesses [19]. Moreover, a review of the psychological issues among individuals with IBD noted that coping strategies are needed to help these individuals manage the symptoms and their psychological impact [39], but they did not outline what those strategies were or what good adjustment entails. We addressed this gap using a mixed-methods design and a large sample of individuals with IBD. Guided by a theoretical framework and previous research, we developed three hypotheses:

- Hypothesis 1 (H1): As living with a chronic illness can be a traumatic and life-changing experience, we expected that the majority of participants with IBD would ask *Why me*?
- Hypothesis 2 (H2): With no known cause for IBD and research showing that meaning-making explanations are multidimensional, we expected that participants who answered the *Why me*? question would report a variety of meanings for their illness.
- Hypothesis 3 (H3): As answering the *Why me*? question has been associated with psychological adjustment, we expected that participants who made meaning would report optimal psychological adjustment (i.e., higher levels of acceptance, subjective well-being, and PTG) than those still searching or who had not found meaning.

## Materials and methods

### Ethics statement

The University of Alberta’s research ethics board reviewed and approved this study (Pro00113880). All participants provided their informed consent to take part in the survey by clicking an “I consent” button after reading through the consent form.

### Research design

We conducted an online survey that used a mixed-methods design [40]. Using quantitative and qualitative methods in a single-phased study allowed us to better understand the research questions and to generate integrated knowledge on a topic in which little is known.

### Procedure

Between October 1 2021 to April 30 2022, we used convenience sampling to recruit participants through notices posted to online groups (e.g., Facebook) for individuals with IBD. Individuals interested in participating clicked a link that took them to the study website where they read the consent form. Once they indicated their consent by clicking an “I consent” button, the study could begin. Participants completed the demographic portion of the survey first, followed by questions about meaning making, and scales to assess acceptance, subjective well-being, and PTG. Participants were not compensated.

### Participants

There were 331 individuals who completed our survey. According to a power analysis using G*Power [41], we needed 235 participants for a medium effect size using .05 alpha level. For inclusion criteria, participants needed a medical diagnosis of IBD and to be 18 years or older. We removed 14 individuals because they reported having irritable bowel syndrome (*n* = 6) or had excessive missing data (*n* = 8). Our final sample included 317 participants, of which 205 (64.7%) had Crohn’s disease, 90 (28.4%) had ulcerative colitis, and 22 (6.9%) had collagenous colitis or lymphocytic colitis. Years since diagnosis was 10.1 years (*SD* = 9.1). Participants were approximately 31 years old (*SD* = 10.3), and mostly female (*n* = 230, 72.6%). The sample was not ethnically diverse, as most participants identified as White (*n* = 274, 86.4%), followed by Black/African American (*n* = 18, 5.7%), multiracial (*n* = 17, 5.4%), and Asian (*n* = 8, 2.5%). The majority had a college or university education or more (*n* = 162, 51.1%). Most were working full-time (n = 152, 47.9%), part-time (*n* = 60, 18.9%), not currently working (*n* = 48, 15.1%), or retired (*n* = 5, 1.6%).

### Measures

Participants completed a survey containing demographics and disease severity, questions about meaning making, and four validated scales to measure psychological adjustment.

#### Demographics

We gathered information about age, gender, ethnicity, education, and employment status. We asked disease-related questions, including the type of IBD they were diagnosed with, month/year diagnosed, and medications. Questions about their disease provided information about our sample and served to screen out those without a medical diagnosis.

#### Disease severity

The Short Inflammatory Bowel Disease Questionnaire [42] measured disease-specific quality of life within the past two weeks. The 10 items were rated on a 7-point scale from 1 (*all the time*) to 7 (*never*). Total scores were summed, and higher values indicated less disease activity. The scale has been used in clinical settings whereby patients with scores ≥ 50 are considered to be in clinical remission [43]. The scale has good reliability with a Cronbach alpha value of 0.90 [42].

#### Meaning making

We developed two questions based on previous research on meaning making [34, 44]. The first question assessed if participants had searched for meaning, and asked, “Individuals who have been diagnosed with a chronic disease might find themselves thinking about why they were diagnosed with it. Have you ever asked ‘*Why me*?*’”* (yes/no). For those who answered ‘yes’, a second question assessed how they had made meaning of their illness, and asked, “Thinking about IBD, why do you think you were diagnosed with it?” While participants could provide causal attributions, including *Why me*? in the question encouraged participants to think about why this adversity happened to them and not others, which can give the researchers a better understanding of the participants’ assumptions about control and fairness [34]. Using the responses to these two questions, we coded participants into one of three groups: those who asked *Why me*? and provided an explanation (found meaning group), those who asked *Why me*? but did not have an answer yet (still searching group), and those who never asked *Why me*? or were not searching for meaning (no meaning/not searching group).

#### Acceptance

We assessed acceptance using the two-item acceptance subscale of the Brief COPE survey [45], which measures strategies for coping with stressors. Participants responded using a 4-point scale from 1 (*I haven’t been doing this at all*) to 4 (*I’ve been doing this a lot*). A subscale score was averaged, and higher values indicated more acceptance. The scale showed good reliability with a Cronbach’s alpha coefficient of .67.

#### Positive and negative affect

The Positive and Negative Affect Schedule [46] consists of 10 words describing positive feelings (e.g., inspired, excited) and 10 words describing negative feelings (e.g., ashamed, nervous). Participants indicated the extent to which they felt this way over the past week using a 5-point scale from 1 (*very slightly or not at all*) to 5 (*extremely*). The subscale scores were summed, with higher scores representing higher levels of either Positive Affect or Negative Affect. The scale shows good reliability with Cronbach’s alpha coefficients ranging from 0.86 to 0.90 [47]. We found good Cronbach’s alpha coefficients for Positive Affect at .90 and Negative Affect at .89.

#### Satisfaction with life

The Satisfaction with Life Scale [37] is a five-item scale measuring global judgments about one’s life satisfaction. Participants responded using a 7-point scale from 1 (*strongly disagree*) to 7 (*strongly agree*). Scores were summed. While values were interpreted as a continuous variable, benchmark scores were examined: 31–35 indicated being extremely satisfied, 26–30 indicated being satisfied, 21–25 indicated being slightly satisfied, 20 indicated being neutral, 15–19 indicated being slightly dissatisfied, 10–14 indicated being dissatisfied, and 5–9 indicated being extremely dissatisfied [48]. The SWLS had good reliability with a Cronbach’s alpha coefficient of .90.

#### Posttraumatic growth

The Posttraumatic Growth Inventory (PTGI) [32] is a 21-item scale that assesses personal growth following a life crisis. The PTGI consists of five subscales: relating to others (seven items), new possibilities (four items), personal strength (three items), spiritual change (two items), and appreciation of life (five items). Participants responded to items using a 6-point scale from 0 (*did not experience this change*) to 5 (*experienced this change to a very great degree*). Scores for each subscale were summed. While a global PTG score is often reported, research examining the PTGI’s factor structure suggests the five-factor model is the best-fitting model [10]. The PTGI showed good reliability for each subscale, with Cronbach’s alphas coefficients ranging from .84 to .89.

### Data analysis

Using SPSS version 26, we calculated the percentage of participants who reported answering *Why me*?, and the means and standard deviations of the outcome variables. We used analysis of variance (ANOVA) to compare demographic differences on the outcomes, as well as correlations to explore the relationships between the variables. For the open-ended, meaning-making question, we analyzed the data using Braun and Clarke’s [49] six-step thematic analysis approach: (1) familiarization (e.g., reading and re-reading the response and noting ideas); (2) identifying codes (e.g., identifying phrases and terms relevant to the topic); (3) developing themes (e.g., moving codes into potential themes); (4) reviewing themes (e.g., examining themes to ensure they align with the content we extracted); (5) defining themes (i.e., describing each theme and giving is a label), (6) producing a report (e.g., presenting findings about the research question). Coding was completed independently by two trained research assistants and the first author. For each theme identified, we reviewed how many participants contributed to it. While such counts are not commonly used in thematic analysis [49], we wanted to provide additional information about each theme’s frequency. We assessed coding consistency by double-coding 75% of the responses, and found an inter-coder reliability of 93%. We resolved coding disagreements through consensus.

To determine whether the three meaning-making groups (i.e., no searching, still searching, meanings made) differed on psychological adjustment (e.g., acceptance, positive affect, negative affect, satisfaction with life, and PTG), we conducted a profile analysis. A profile analysis is the multivariate equivalent of a repeated-measures MANOVA. Prior to analysis, we examined the data to ensure assumptions were met. Our screening revealed that there were no univariate outliers. Several instances of missing data were addressed using mean substation [50]. We found no multivariate outliers, which we checked using the Mahalanobis distance test at alpha = .01. The data were normally distributed according to skewness and kurtosis levels. As profile analysis requires the outcome variables to be measured on the same scale, we transformed the scores to *z*-scores [50]. We used a Bonferroni correction on all follow-up analyses (i.e., one-way ANOVAs, simple effects) to control for familywise error. The masked data has been made publicly available through Scholars Portal Dataverse and can be accessed at https://doi.org/10.5683/SP3/CA2MJS.

## Results

### Descriptive statistics

The means, standard deviations, and correlations for the outcome variables are presented in Table 1. As shown, participants reported moderate levels of positive affect, slightly lower levels of negative affect, and were slightly satisfied with life. The global PTG score was 52.26 (*SD* = 23.88), which was similar to what has been previously reported for individuals with arthritis (*M* = 50.20, *SD* = 25.92) or IBD (*M* = 54.95, *SD* = 23.80) [10]. The scores of each PTG subscale were also similar to those reported by Taku et al. (2008) and Purc-Stephenson (2014). Women reported greater perceptions of relating to others (*M* = 11.09, *SD* = 6.82) than men (*M* = 9.69, *SD* = 5.52), *F*(1, 316) = 5.14, *p* < .05.

**Table 1:** Mean and standard deviations for the outcome variables.

Variable	Mean (*SD*)
Acceptance	3.25 (0.72)
Positive affect	29.08 (7.50)
Negative affect	23.23 (8.05)
Satisfaction with life	21.12 (8.09)
Relating to others	18.56 (9.03)
New possibilities	10.85 (6.63)
Personal strength	11.86 (5.26)
Spiritual change	2.97 (3.30)
Appreciation of life	8.43 (4.31)

We explored potential demographic differences across the outcome variables. The correlations are presented in Table 2. We found that age was negatively correlated with new possibilities (*r* = -.21, *p* < .01) and greater illness severity was correlated with higher levels of positive affect (*r* = .21, *p* < .01), lower affect (*r* = .36, *p* < .01), and fewer perceptions of personal strength (*r* = .13, *p* < .05). No other demographics or illness severity variables were significantly related to the outcome variables.

**Table 2:** Correlation coefficients for the outcome variables.

Variable	1	2	3	4	5	6	7	8	9
1. Acceptance	--								
2. Positive affect	.28**	--							
3. Negative affect	-.21**	-.24**	--						
4. Satisfaction with life	.25**	.51**	-.43**	--					
5. Relating to others	.19**	.37**	-.03	.32**	--				
6. New possibilities	.19**	.47**	-.07	.32**	.62**	--			
7. Personal strength	.28**	.46**	-.14*	.35**	.53**	.61**	--		
8. Spiritual change	.18*	.14*	-.06	.11*	.43**	.46**	.43**	--	
9. Appreciation of life	.17*	.37**	.04	.22**	.57*	.59**	.51**	.41**	--

*Note*. *N* = 317.

*p < .05.

**p < .001

### Answering Why Me?

Approximately 85% (*n* = 269) of participants reported asking *Why me*? A one-way ANOVA showed that participants who reported experiencing more symptom distress (*M* = 46.82, *SD* = 14.84) were more likely to report asking *Why me*? than participants who reported experiencing less symptom distress (*M* = 52.02, *SD* = 13.58), *F*(1, 316) = 5.11, *p* < .05. We also found that age mattered. A one-way ANOVA revealed that younger participants (*M* = 30.38, *SD* = 10.05) were more likely to ask *Why me*? than older participants (*M* = 35.94, *SD* = 10.18), *F*(1, 316) = 7.39, *p* < 01. These results supported H1 that symptom distress would motivate the majority of individuals to ask *Why me*? We also explored possible gender differences to determine if men or women asked *Why me*? at different rates. The results were not significant, χ^2^ (1, 317) = 0.02, *p* > .05, whereby men and women asked the Why me? question at similar rates, 85.5% and 84.7%, respectively.

### Ways of meaning making

Nearly 60% of participants had made meaning (*n* = 122) or were still searching for meaning (*n* = 67) in their illness experience; the remaining were not searching or did not find meaning (*n* = 128). Focusing on those who had made meaning, our thematic analysis revealed five meaning-making themes: Catalyst for Personal Growth, Self-Blame, Divine Intervention, Genetics, and Chance. Our findings supported H2, which stated that the meaning-making explanations would be multi-dimensional. The themes are described in Table 3 with verbatim quotes from our participants. We also conducted a series of one-way ANOVAs to determine if the scores on any outcome were related to the type of meaning made. We found no significant differences, which suggested that psychological adjustment is not dependent on the type of explanation made about one’s illness.

**Table 3:** Meaning-making themes, descriptions, and example quotes.

Theme	Description	Example Quotes
Catalyst for Personal Growth	Their illness was meant to trigger a positive change in their lives, with many referring to their illness as a starting point for a healthier lifestyle or stimulating character growth such as maturity, independence, and strength.	I try to compare this experience so far with that of my peers, and imagine how this will shape my character in the future. (Participant 323)
*n* = 54		I answer this by telling myself that this experience has prepared me for dealing with many other more difficult experiences and has allowed me to better understand the pain of other people. (Participant 51)
Self-Blame	Their illness was understood as karmic punishment for unhealthy life choices they made before diagnosis, or even something they did in a past life. These individuals viewed their illness negatively and often believed they could have prevented it.	Sometimes I think, that maybe I did something in a past life that I now have to atone for. (Participant 372)
*n* = 28		I question sometimes have I done something in the past that I am now paying the price for like karma as well as living an unhealthy lifestyle during my teens and early 20’s (Participant 87)
Divine Intervention	Their illness was a part of a divine intervention or God’s plan. While they may not understand the reason why they were diagnosed, they have faith that a divine plan was created for them and this was merely a necessary step towards who and where they are meant to be.	I believe that I survived everything I went through when I developed ulcerative colitis to be of help to others with ileostomies and because God wanted me to do something special with my life. (Participant 112)
*n* = 24		I believe only special people are given these specific illnesses because God knows they can handle it… and for me to help people by spreading the knowledge and support that I have. (Participant 183)
Genetics	Their illness was understood as a genetic predisposition and there was nothing to change that. Participants did not seek a deeper meaning, except to mention other family members who suffer from similar illnesses which has created a collective experience.	Having an uncle with UC, a brother with proctitis and a daughter with Crohn’s I think genetics definitely have played a part in it. (Participant 238)
*n* = 11		The problem is genetic. My mother’s family carries the gene for it. It is part of life. (Participant 10)
Chance	Their illness was understood as random chance or bad luck. These participants removed personal blame because they believed they had no control in preventing their diagnosis.	Illness happens to almost everybody rather randomly. (Participant 259)
*n* = 5		If you are trying to figure out if I think there is some karma or spiritual connection to my illness, I don’t really feel one …It is simply odds that I have this illness. (Participant 74)

### Meaning making and psychological adjustment

Table 4 presents the means and standard deviations of the psychological outcomes for the three groups. The profile analysis revealed a significant multivariate group x outcome interaction, Wilks Δ = .85, *F*(7,14) = 4.44, *p* < .0001. This interaction signified that the profiles of the three groups differed from each other across the set of outcomes.

**Table 4:** Means, standard deviations, and F tests for the three meaning-making groups.

Variable	No Searching		Still Searching		Meanings Made		*F*(2, 314)	Ƞ^2^
	M	SD	M	SD	M	*SD*		
Acceptance	3.16	0.76	3.24	0.66	3.34	0.70	1.924	.010
Positive affect	26.96	7.60	28.97	6.83	31.35	7.13	11.41***	.068
Negative affect	22.83	8.12	26.01	8.56	22.13	7.36	5.46**	.034
Satisfaction with life	20.84	8.29	19.34	8.07	22.39	7.74	3.23*	.020
Relating to others	15.65	9.51	19.13	7.96	21.31	8.14	13.44**	.079
New possibilities	8.73	6.32	10.93	6.31	13.02	6.45	14.12***	.083
Personal strength	10.68	5.69	11.46	4.88	13.31	4.65	8.43***	.051
Spiritual change	2.06	2.85	3.07	3.41	3.87	3.47	9.88***	.059
Appreciation of life	6.99	4.30	9.25	3.92	9.47	4.14	12.81***	.075

*Note*. *N* = 317.

* *p* < .05.

**p < .01.

***p < .001.

**Fig 1:**
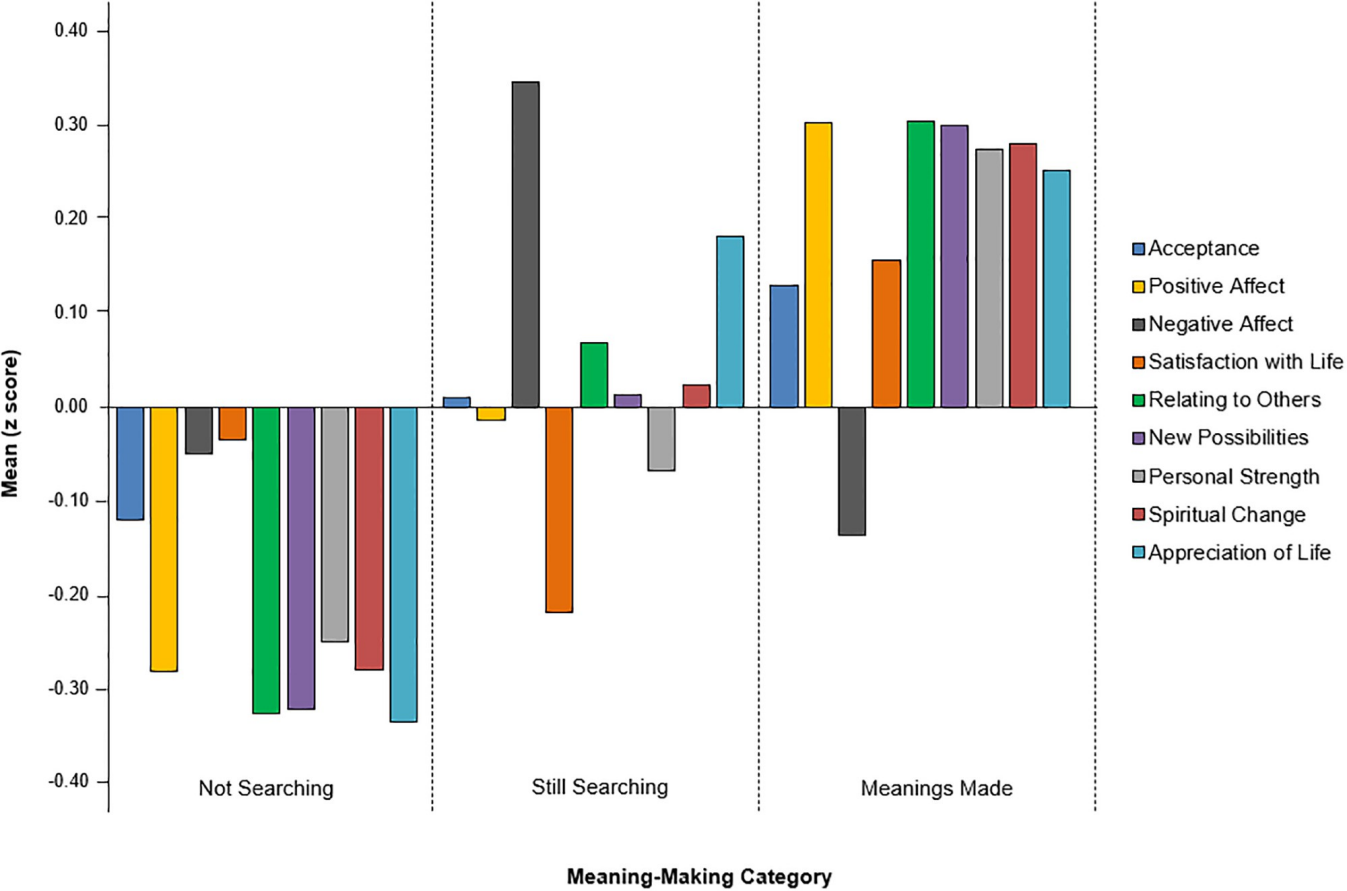
The profiles of each meaning-making group.

To understand how the three groups differed from each other, we first focused on *each outcome* separately using a series of one-way ANOVAs. As Table 4 shows, we found significant group differences for each outcome (all *p*s < .05), except for acceptance. We conducted pairwise comparisons for all significant group differences. For positive affect, the meanings-made group reported significantly higher scores than the no-searching group (*p* < .001). For negative affect, the no-searching and still-searching groups reported significantly higher scores than the meanings-made group (both *p*s < .05). For SWL, the meanings-made group reported higher scores than the still-searching group (*p* < .05). For the PTGI subscales, the meanings-made group reported significantly higher scores than the no-searching group on each subscale (all *p*s < .001). The still-searching group reported significantly higher scores than the no-searching group on relating to others (*p* < .05), appreciation of life (*p* < .001). Fig 1 presents the z scores of the three groups on each outcome.

Next, we focused on the profiles of *each group separately* to determine how each differed across the outcome variable using simple effects. The no-searching group reported neutral satisfaction with life scores (*M* = 20.84, *SD* = 8.29) and little amounts of negative affect (*M* = 22.83, *SD* = 8.11). However, Fig 1 shows that they perceived very little PTG or positive affect. Results showed significantly lower positive affect, relating to others, new possibilities, spiritual change, and appreciation of life scores (all *p*s < .05) compared to satisfaction of life scores. Likewise, they reported significantly lower relating to others, new possibilities, spiritual change, and appreciation of life scores compared to negative affect (all *p*s < .05).

The still-searching group reported some positive affect and acceptance of IBD, but a high degree of negative affect, little satisfaction with life, and some PTG. Fig 1 shows that they reported more negative affect than positive affect (*p* < .05). They also reported being slightly dissatisfied with life (*M* = 19.34, *SD* = 8.07), but perceived significantly more PTG in relating to others (*p* < .05) and appreciation of life (*p* < .01). Comparing the PTGI subscales, they reported some PTG in all areas except perceptions of personal strength (*p* < .05).

In contrast, the meanings-made group showed optimal adjustment with low levels of negative affect and high levels of positive affect, satisfaction with life and perceived PTG. As shown in Fig 1, participants reported significantly higher levels of positive affect (*p* < .001), satisfaction with life (*p* < .05), acceptance (*p* < .001), and perceived personal growth on each PTGI subscale (*p* < .01) compared to negative affect. These results support H3 that stated participants who had made meaning would show optimal adjustment compared to those still searching for meaning or those who were not searching for meaning.

## Discussion

Our study investigated meaning making among adults with IBD and its impact on illness acceptance, subjective well-being, and PTG. As expected, being diagnosed with IBD was associated with asking *Why me*?, with 85% of our 317 participants asking themselves this question. Consistent with the assumptions of the meaning-making model, asking *Why me*? was most common among participants who experienced greater illness distress than those who could be categorized as being in clinical remission. Yet, despite the high percentage of those asking *Why me*?, only 39% provided an explanation and 21% were still searching for meaning.

Although we assessed meaning making similar to previous research, our findings differ from what has been reported. For example, previous research found that 18% of individuals with tinnitus provided an answer to *Why me*? [34] whereas others reported that 78% of cancer patients reported an explanation [17]. In support of H1 and consistent with growth theories [30–32], a search for meaning can be triggered by a shattering of assumptions especially when there is a significant threat to an individual’s well-being. Tinnitus, IBD, and cancer may be associated with different threats to well-being, with tinnitus presenting little threat, cancer presenting a significant threat, and IBD somewhere in the middle.

Individuals with IBD provided a variety of explanations for their illness, which supported H2. The most common meaning-making theme was Catalyst for Growth, whereby participants believed IBD was meant to trigger a positive change in their lives. This theme was also most common among individuals with multiple sclerosis and their caregivers [14, 21]. For example, individuals with multiple sclerosis perceived their illness as an opportunity for personal growth by opening new doors, allowing them to develop greater self-awareness, and to make healthier lifestyle choices [14].

It was also common for participants to blame themselves for their illness. Self-blame is a common attribution in research on other health conditions [4, 51]. For example, Kalla et al. [51] interviewed eight individuals with a chronic illness and found that participants believed their illness resulted from unresolved past traumas, drug habits, or from pushing themselves too hard in pursuit of goals. When we compared the meaning-making themes, we found no differences in the outcome variables. Thus, our results are consistent with previous research on cancer patients that finding meaning is important, and reinforces the notion that the specific type of meaning made does not influence adjustment.

Consistent with our hypotheses and in support of the meaning-making model, those who found meaning also reported accepting their illness, greater subjective well-being, and PTG than those still searching or had not found meaning/were not searching. Our findings support a growing body of research showing that meaning making in chronic illness is associated with positive outcomes [14, 24, 25, 34, 35]. For example, Davis and Morgan [34] reported that individuals with tinnitus were able to answer *Why me*? were more likely to report positive changes than those who reported not asking that question (49.1% vs. 28.2%) and marginally more likely than those not yet able to answer that question (49.1% vs. 35.4%).

Contrary to our expectations, failure to make meaning did not necessarily relate to poor psychological adjustment. While participants in the no-meaning group reported little PTG, they reported similar levels of acceptance, satisfaction with life, and negative affect as individuals who had made meaning. In contrast, those still searching for meaning reported more negative affect and less satisfaction with life compared to the other two groups, yet perceived some PTG in all domains except personal strength. In our study, those still searching for meaning reported marginally more symptom distress than the other groups. Thus, those still searching for meaning may have been experiencing more stressful situations (i.e., continuous flares) than the other groups, which created a greater discrepancy between their global and situational meanings [4]. However, some researchers argue that thinking about adversity induces unproductive rumination that impedes adjustment [52].

Consistent with previous research [22, 35], meaning-making profiles were associated with different levels of adjustment and PTG. For example, those still searching for meaning represented the most maladaptive profile as they reported many depressive symptoms and low levels of life satisfaction. In contrast, individuals who had found meaning and were no longer searching for meaning represented the optimal profile as they reported few depressive symptoms and greater levels of life satisfaction; those who had not found meaning and were not searching also showed few depressive symptoms.

Our finding that meaning making was associated with psychological adjustment contributes to a growing body of research showing that making sense of adversity is related to better psychological health [20, 24, 35]. Rehabilitation counselors working with individuals with a chronic illness could integrate meaning making into therapeutic conversations. Helping an individual think through their illness experience to find meaning may be a way to develop PTG. Furthermore, as individuals with IBD face physical, psychological, and social challenges throughout their life, counselors could educate their clients on self-management strategies that support meaning making such as self-analysis (i.e., expressive writing) and self-disclosure (i.e., sharing and talking about adversity) [32]. Also, self-disclosure may benefit individuals with chronic illnesses. For example, a systematic review of 13 qualitative studies on online peer-to-peer support for individuals with chronic illness [53]. They reported that online groups provided a tool for reading, posting, and learning about illness experiences, as well as a safe space to express explicit details about the disease. While online groups provide mutual solidarity and emotional support, they can also be a powerful platform to help individuals reframe negative emotions into something positive and find meaning in illness.

We recruited participants online, which means we could not confirm a medical IBD. However, we reviewed the pattern of responses to questions about IBD and medications to screen out individuals. Also, we used a convenience sample that increases the likelihood of a self-selection bias. To minimize this bias, we offered no incentive to complete our study, and we recruited from a broad range of online groups. Moreover, recruiting participants allowed us to include individuals with a range of illness severity who may not have had easy access to in the community. In fact, samples recruited from the internet often have more health issues and poorer quality of life than those recruited from medical clinics [54]. Despite this, our sample was mostly female and White, so our results may not generalize to other groups or geographical contexts.

Our results underscore the need to assess a comprehensive set of outcomes when evaluating adjustment to adversity. We found that perceptions of PTG do not necessarily reflect high levels of subjective well-being or acceptance. Future research needs to test the relationship between meaning making and psychological adjustment to determine how it changes across time. Also, as this is the first study to explore meaning making among individuals with IBD, current interventions for this patient population are likely uninformed by meaning making. Research on meaning-oriented therapy among individuals with a chronic disease [51], as well as those who have lost a loved one [55], demonstrates success at facilitating making sense of adversity. Counsellors need to explore if these interventions also promote psychological or physical well-being among their patients.

Living with a chronic illness such as IBD is challenging in that the symptoms may fluctuate or worsen over time, which necessitates counselors support their clients to learn ways to cope with a changing identity, sense of purpose, and meaning. Our findings contribute to a growing body of research highlighting that finding meaning in the illness experience plays an important role in coping with trauma and adversity [20, 24, 35]. Our findings provide counselors and other healthcare providers a greater understanding of the ongoing stressors individuals with IBD encounter, and the role of meaning making on rates of accepting an illness, subjective well-being, and personal growth.
